# Prevalence and Risk Factors of Aphthous Ulcers Following Periodontal Surgery: A Cross-Sectional Analysis

**DOI:** 10.3390/jcm15062237

**Published:** 2026-03-15

**Authors:** Sultan Albeshri, Raed Alrowis, Nouf AlAkeel, Mazen Almobarki, Ibrahim S. Alsanie, Razan Alaqeely

**Affiliations:** 1 Department of Periodontics and Community Dentistry, College of Dentistry, King Saud University, Riyadh 11545, Saudi Arabia; salbeshri@ksu.edu.sa (S.A.);; 2 Department of Oral Medicine and Diagnostic Sciences, College of Dentistry, King Saud University, Riyadh 11545, Saudi Arabia

**Keywords:** aphthous ulcers, periodontal surgery, risk factors, CHAID analysis, post-operative complications, oral mucosa

## Abstract

**Background/Objectives:** This study aimed to determine the prevalence of aphthous ulcers following periodontal surgery and to identify demographic, behavioral, and clinical predictors of ulcer history before surgery and ulcer development after surgery. **Methods:** A cross-sectional study was conducted among 227 adult patients undergoing periodontal surgical procedures between November 2024 and May 2025. Demographic, medical, behavioral, and oral health data were collected. Postoperative follow-up at 1 and 2 weeks included a standardized clinical assessment of aphthous ulcers. Statistical analyses included descriptive statistics, chi-square tests, and Chi-squared Automatic Interaction Detection (CHAID) decision tree modeling. **Results:** Aphthous ulcers developed in 47 patients (20.7%), predominantly within the first postoperative week. CHAID analysis identified age, marital status, and smoking as predictors of preoperative ulcer history (classification accuracy: 73.6%), whereas age and family history predicted postoperative ulcer development (79.4%). Periodontal procedure type was significantly associated with postoperative medication prescription (χ^2^ = 300.45, *p* < 0.001), suture selection (χ^2^ = 69.19, *p* = 0.024), and ulcer number (χ^2^ = 48.43, *p* = 0.031), but not ulcer size or anatomical location. Most ulcers were minor and primarily involved the buccal mucosa. **Conclusions:** Postoperative aphthous ulceration is a common complication of periodontal surgery, affecting approximately one-fifth of patients. Distinct risk profiles for pre- and post-surgical ulceration highlight the roles of patient-related susceptibility and surgical complexity. These findings support the use of structured risk stratification to guide preoperative counseling and targeted postoperative management.

## 1. Introduction

Oral aphthous ulcers, also known as canker sores, are among the most common mucosal diseases of the oral cavity, with a reported lifetime prevalence of 5–66% [1,2,3]. They are painful, recurrent lesions affecting non-keratinized oral mucosa, including labial and buccal mucosa, the ventral tongue, the soft palate, and the floor of the mouth [4,5]. Although typically self-limiting, aphthous ulcers can significantly impair quality of life due to interference in the basic functions of life, i.e., eating, speaking, or oral hygiene maintenance [6,7].

Clinically, aphthous ulcers are classified into minor (80–85%), major (10–15%), and herpetiform (5–10%) subtypes based on size, depth, and healing characteristics [8,9].

The causes and mechanisms of recurrent aphthous stomatitis (RAS) are complex and multifactorial, with interactions among genetic predisposition, immunological dysregulation, environmental factors, and local traumatic factors [10]. A Th1-dominant immune response characterized by increased IFN-γ, IL-2, and IL-12 has been demonstrated in aphthous lesions, along with inflammatory epithelial degeneration [11,12]. Recent advances in the molecular pathophysiology of RAS have identified several key mechanisms. It has been shown that interferon-gamma (IFN-γ), interleukin-2 (IL-2), and interleukin-12 (IL-12) are increased in the lesions of RAS, which is a characteristic of a Th1-dominant immune reaction [3]. Alterations in the oral microbiome, particularly increased Streptococcus species and reduced protective commensals, have also been implicated [13,14]. Familial clustering and higher concordance among first-degree relatives support a genetic component [15,16]. Behavioral and environmental factors, including psychological stress, nutritional deficiencies, hormonal variation, food sensitivities, and mechanical mucosal trauma, further contribute to disease expression [2,17,18,19]. It is also worth noting that psychological stress has been recognized as a trigger and modifying factor of RAS and likely acts in several ways, including heightened mucosal sensitivity to trauma, decreased salivary flow, conditioning of parafunctional behaviors such as bruxism, impaired immune regulation, and augmented inflammatory vulnerability [17,20]. Recent studies using validated stress assessment scales have established a strong association between perceived stress levels and the frequency and severity of RAS [21,22].

Periodontal surgery encompasses a range of invasive procedures that disrupt the oral mucosal barrier and transiently modify local immune responses through tissue trauma, flap elevation, suturing, and postoperative inflammation [23] Surgical and traumatic oral interventions have been associated with the onset of aphthous ulceration; however, evidence specifically linking periodontal surgical procedures to postoperative aphthous ulcers remains limited [24] To date, no study has systematically examined the prevalence, risk factors, and clinical characteristics of aphthous ulcers specifically following periodontal surgery using standardized diagnostic criteria and advanced analytical methods.

The main research question that guided this study was: What is the incidence of aphthous ulcers that occur post-periodontal surgery, and what predicts their occurrence? To answer this question, we conducted the study with the following objectives: (1) the factors, which are related to the history of ulcers before surgery, (2) predictors of the emergence of ulcers in the postoperative period, and (3) the connection between the type of surgical procedure, clinical management decision-making, and the outcomes of ulceration. Accordingly, three hypotheses were tested: (H1) demographic and behavioral factors predict preoperative ulcer history; (H2) age and family history predict postoperative ulcer development; and (H3) procedure type is associated with clinical management strategies and ulceration outcomes.

This study offers several novel contributions to the literature: (1) it provides the first precise prevalence estimate of postoperative aphthous ulcers in a periodontal surgery cohort; (2) it uses CHAID decision tree analysis to identify distinct risk profiles for pre-existing RAS versus surgically precipitated ulcers; (3) it demonstrates that surgical complexity influences ulcer number but not severity; and (4) it develops a clinically applicable risk stratification approach for preoperative counseling.

## 2. Materials and Methods

### 2.1. Study Design and Setting

This cross-sectional observational study was conducted at the Department of Periodontics, Dental University Hospital, King Saud University, between November 2024 and May 2025. The study adhered to STROBE guidelines for cross-sectional studies [25] and was approved by the Institutional Review Board (E-24-9319).

### 2.2. Study Population and Sampling

Adult patients (≥18 years) requiring periodontal surgical treatment were consecutively enrolled. A total of 227 participants met the eligibility criteria and provided informed consent. Inclusion criteria comprised planned receipt of a periodontal surgical procedure, absence of active oral ulcerations or mucosal lesions, and ability to attend postoperative follow-up. Exclusion criteria included immunodeficiency disorders; use of systemic corticosteroids or immunosuppressive agents; active systemic infection or hospitalization within the preceding 3 months; autoimmune diseases with oral manifestations; active malignancy or ongoing chemotherapy or radiotherapy; known allergies to medications commonly used in periodontal surgery; and inability to attend follow-up visits.

### 2.3. Data Collection Procedures

Baseline data were collected during the preoperative visit through structured interviews, review of medical and dental histories, and clinical examination [7,18]. All surgical procedures were performed according to standard clinical protocols, and intraoperative variables and postoperative medications were recorded [23,26]. Postoperative follow-up included assessment of aphthous ulcers using validated clinical criteria for ulcer classification and anatomical localization [5,27].

To ensure accurate distinction between aphthous ulcers and surgically induced traumatic ulcers, the following validated diagnostic criteria were applied: (1) characteristic round or oval shape with an erythematous halo and yellowish pseudomembrane; (2) location on non-keratinized mucosa (buccal mucosa, labial mucosa, ventral tongue, floor of mouth); (3) presence at sites distant from the surgical field; and (4) absence of direct correspondence with suture lines or surgical incisions. Traumatic ulcers were defined as lesions occurring directly on surgical sites, adjacent to sutures, or on keratinized tissue within the surgical field.

### 2.4. Data Quality Control

To ensure data quality and reliability, each examiner participating in the assessment of ulcers was trained in calibration to maintain uniform standards of diagnosis and documentation [25]. Inter-examiner reliability was evaluated using Cohen’s kappa statistic, which yielded a value of 0.89, indicating excellent agreement. A second investigator conducted an independent review of 10 percent of patient records to verify the accuracy and completeness of the data.

### 2.5. Statistical Analysis

The statistical analyses were conducted using IBM SPSS Statistics version 27.0 (IBM Corp., Armonk, NY, USA), with α = 0.05 for all tests. The sample size was determined using G*Power 3.1 (Düsseldorf; Germany). Based on the calculations, a total of at least 220 participants were required with the following parameters: a 95% confidence level, a significance level (alpha) of 0.05, an effect size (ES) of 0.3, and a statistical power of 0.90.

All variables were subjected to descriptive statistics, with categorical variables presented as frequencies and percentages and continuous variables as means, standard deviations, medians, and interquartile ranges. Postoperative aphthous ulcer prevalence was calculated as the percentage of patients who developed at least one ulcer during the follow-up period, with 95% confidence intervals. All associations among the periodontal procedure type, post-surgical clinical management variables, and oral ulceration outcomes were analyzed using chi-square tests of independence [28]. The CHAID decision tree analysis, a nonparametric algorithm, was used to predict oral ulcer history prior to procedures and the development of oral ulcers following procedures [29,30]. It was selected over traditional logistic regression for the following reasons: (1) it automatically identifies and models non-linear relationships and interactions without requiring pre-specification; (2) it handles categorical predictors without assuming linearity; (3) it produces clinically interpretable visual decision rules that identify specific patient subgroups at risk; (4) it accommodates missing data through surrogate splits; and (5) it mirrors clinical decision-making by sequentially partitioning patients based on predictive factors. The analysis was performed using the CHAID algorithm with a maximum tree depth of 3, a minimum parent node size of 100, and a minimum child node size of 50 [29,30].

No internal or external validation was performed, which may result in overfitting and overestimation of predictive accuracy; therefore, these models require validation in independent cohorts before clinical implementation.

## 3. Results

The final analytical sample included 227 patients, predominantly middle-aged adults. The largest age group was 36–45 years (22.9%), followed by 26–35 years (19.8%) and 56–65 years (19.4%), while participants aged ≥65 years comprised the smallest proportion (9.7%). Females represented 57.7% of the sample. Most participants were married (77.1%), Saudi nationals (92.5%), and either had no formal education (41.0%) or a university degree (33.0%). Unemployment was the most common employment status (45.8%).

### 3.1. Clinical and Behavioral Profile

Most participants (80.2%) reported no systemic medical conditions and were not taking medications at baseline (79.3%). Among reported conditions, hypertension (5.3%) and type II diabetes mellitus (4.4%) were most prevalent. Table 1 shows the complete medical history and medication profile of the study population. Although the majority (80.2%) had no reported systemic medical condition, the commonest ones were hypertension (5.3%), type II diabetes mellitus (4.4%), hypothyroidism (2.2%), and asthma (1.8). Antihypertensives were most frequent among patients taking medications (20.7%) such as beta-blockers (3.1%), ACE inhibitors (2.2%), and calcium channel blockers (1.8%). A family history of oral ulcers was uncommon (2.2%), whereas a personal history was reported by 18.5% of participants. Current smokers comprised 18.5% of the sample, with variable daily consumption.

### 3.2. Periodontal Procedures and Clinical Management

The procedures performed during the periodontal were different. The most common procedures were soft-tissue augmentation (22.0%), implant surgery (22.0%), guided bone regeneration (13.2%), periodontal flap surgery (12.3%), tooth extraction (5.7%), and resective surgery (5.7%). Combined procedures involving two or more interventions accounted for 15% of cases.

Regarding postoperative medications, almost a third of patients (32.2%) received no postoperative medications; others received single-agent or combination regimens: NSAIDs and chlorhexidine mouthwash (22.1%), combined antibiotics and NSAIDs (8.8%), and a variety of combinations (15.0%). Suture materials used included Vicryl (60.8%), followed by Prolene (29.1%), PTFE (8.4%), and Monocryl (1.8%).

### 3.3. Postoperative Ulceration Outcomes

Postoperatively, 20.7% of patients developed oral ulceration. Among those with ulcers, the vast majority (19.8% of the total sample) developed 1–5 ulcers, while only 0.9% experienced 6–10 ulcers. When ulcers developed, they were predominantly minor (3–10 mm in diameter; 17.6% of the total sample), with major ulcers (1–3 cm in diameter) occurring in only 2.2% of cases.

Among those with ulcers, the buccal mucosa was the most frequently affected site (8.8% of the total sample), followed by the labial mucosa (1.8%) and the lateral surface of the tongue (1.3%). Overall, these findings indicate that postoperative ulceration was relatively infrequent and, when it occurred, generally mild.

### 3.4. Predictors of Oral Ulcers Before and After Periodontal Procedures

Two CHAID decision tree models were developed to identify factors predicting patients’ oral ulcer history before procedures and post- surgical ulcer development. Table 2 summarizes the specifications and performance metrics of both models.

#### 3.4.1. Model 1: Predictors of Patient History of Oral Ulcers Before Procedure

**Figure 1:**
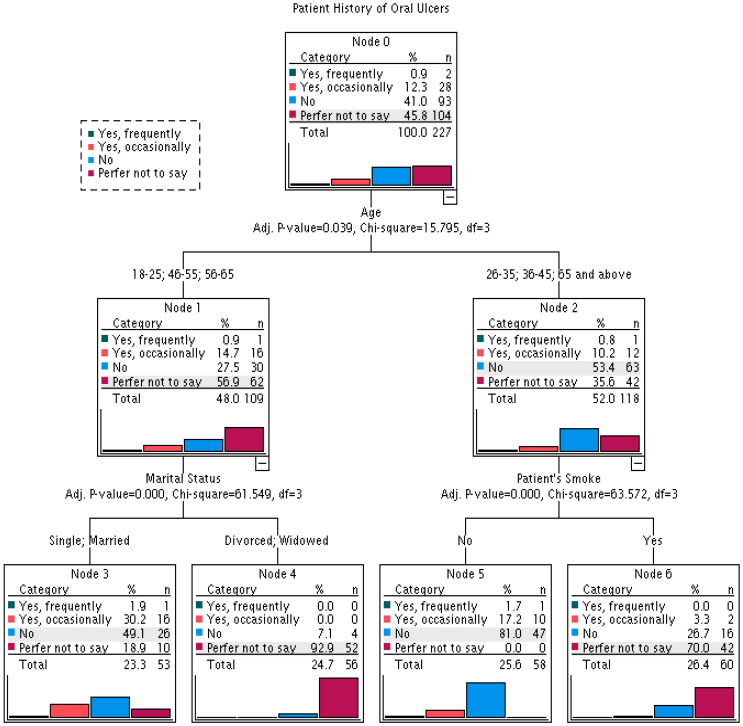
CHAID analysis to identify the risk factors of a patient’s history of oral ulcers before procedure (Model 1).

This CHAID model evaluated predictors of patients’ self-reported history of oral ulcers prior to periodontal surgery (Table 1). Among all variables tested, age, marital status, and smoking status met the inclusion criteria. The final model comprised 7 nodes (4 terminal nodes) with a tree depth of 2. Model performance was moderate, with an estimated risk of 0.264 (SE = 0.029), corresponding to a misclassification rate of 26.4% and an overall classification accuracy of 73.6%. Age was the primary splitting variable, with further stratification by marital status and smoking within specific age groups (Figure 1). These findings support Hypothesis 1, which posits that demographic and behavioral factors significantly predict a history of preoperative ulceration.

#### 3.4.2. Model 2: Predictors of Patient Experience of Ulceration After Procedure

**Figure 2:**
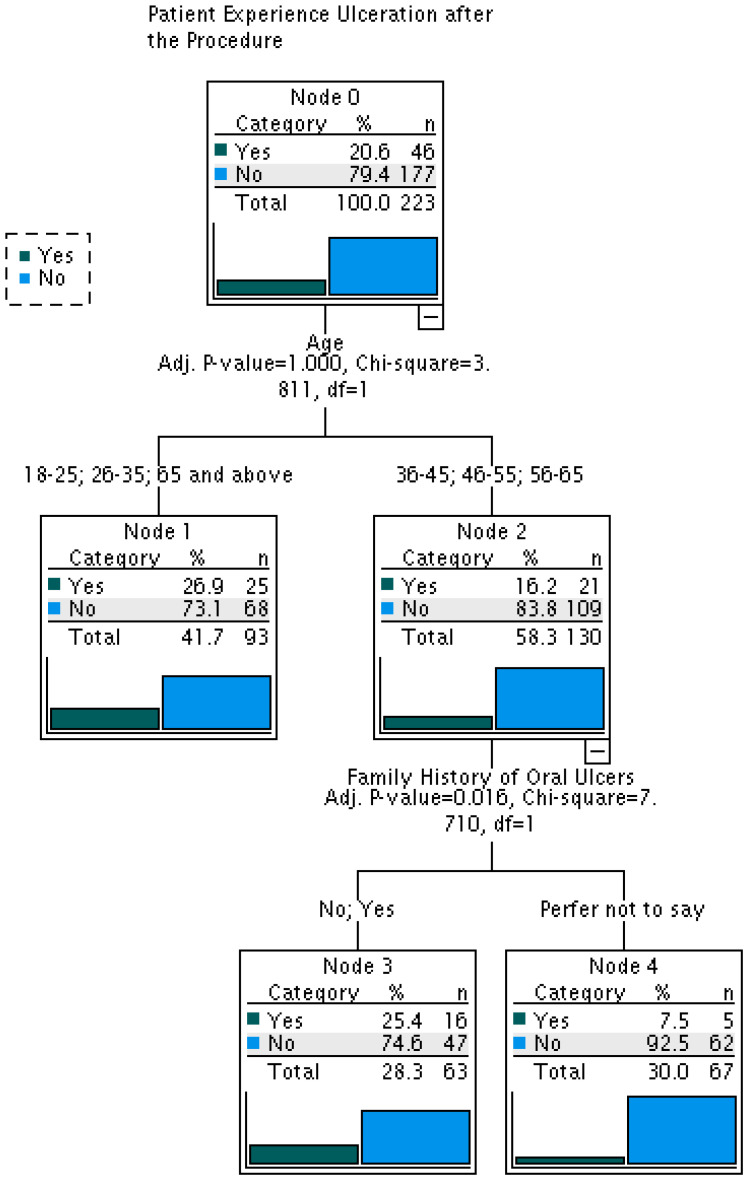
CHAID analysis to identify the risk factors of a patient’s Experience of oral ulceration after the procedure (Model 2).

A separate CHAID model was constructed to identify predictors of postoperative aphthous ulceration (Table 2). Unlike Model 1, this model included only age and family history of oral ulcers as significant predictors, while behavioral factors such as smoking were not retained. The final model was more parsimonious, consisting of 5 nodes (3 terminal nodes) and a tree depth of 2, and demonstrated improved predictive performance. The estimated risk was 0.206 (SE = 0.027), with a misclassification rate of 20.6% and an overall classification accuracy of 79.4%, representing a 5.8% improvement over Model 1. Age was the primary splitting variable, with family history further stratifying risk within age groups (Figure 2), supporting Hypothesis 2.

### 3.5. Comparison of Models and Temporal Patterns

Together, the two models demonstrate distinct predictor profiles across temporal contexts. Preoperative ulcer history was associated with a combination of demographic and behavioral factors, whereas postoperative ulceration was primarily associated with age and familial susceptibility, suggesting a stronger biological influence following surgical intervention.

### 3.6. Association Between Periodontal Procedure Type and Clinical Management and Ulceration Outcomes

Chi-square analyses were conducted to examine whether the type of periodontal procedure was associated with post-surgical clinical management decisions and various oral ulceration outcomes. Results are presented in Table 3 and Table 4.

### 3.7. Medication Prescription After Procedure

A significant association was observed between periodontal procedure type and postoperative medication regimen (χ^2^ = 300.45, *p* < 0.001; Table 2). Less invasive procedures, such as periodontal flap surgery and resective surgery, were more frequently associated with no medication or single-agent therapy, primarily NSAIDs (46.4% and 46.2%, respectively). In contrast, more complex procedures, particularly implant surgery and combined procedures, were associated with multidrug regimens. Implant surgery patients commonly received antibiotics with NSAIDs (20.0%), antibiotics with NSAIDs and chlorhexidine (24.0%), or NSAIDs alone (24.0%), while over half of patients undergoing combined procedures (52.9%) received triple therapy consisting of antibiotics, NSAIDs, and chlorhexidine. Guided bone regeneration also showed higher use of combination regimens, including antibiotics, NSAIDs, and corticosteroids (33.3%).

### 3.8. Suture Type Selection

Suture material selection was significantly associated with procedure type (χ^2^ = 69.19, *p* = 0.024). Vicryl was the most frequently used suture overall (60.8%), particularly in periodontal flap surgery (82.1%), implant surgery (78.0%), and tooth extraction (76.9%). Prolene was more commonly used in resective surgery (53.8%), soft-tissue augmentation (34.0%), and guided bone regeneration (40.0%). PTFE sutures were used more frequently in guided bone regeneration (20.0%), combined procedures (17.6%), and resective surgery (15.4%), while Monocryl was infrequently used (1.8%), limited to soft-tissue augmentation cases.

### 3.9. Ulcer Occurrence and Characteristics

The association between procedure type and ulcer occurrence approached, but did not reach, statistical significance (χ^2^ = 25.27, *p* = 0.065). Combined procedures demonstrated the highest ulcer incidence (44.1%), whereas single procedures showed lower rates (0–25.0%). A significant association was found between procedure type and the number of ulcers (χ^2^ = 48.43, *p* = 0.031), with combined procedures showing the highest proportion of patients developing 1–5 ulcers (41.2%). Development of more than five ulcers was rare, occurring in only two patients.

No significant associations were observed between procedure type and ulcer size (χ^2^ = 33.26, *p* = 0.406) or anatomical location (χ^2^ = 168.61, *p* = 0.642). Across all procedures, ulcers were predominantly minor (3–10 mm), and the buccal mucosa was the most frequently affected site.

Hypothesis 3 was fully supported for clinical management outcomes, with significant associations observed for medication prescription and suture selection. It was partially supported for ulceration outcomes, as procedure type was significantly associated with ulcer number but not with ulcer occurrence, size, or anatomical location.

## 4. Discussion

This study examined the risk factors and clinical characteristics of aphthous ulcers following periodontal surgery and provides findings with direct clinical relevance. Approximately 20.7% of patients developed postoperative ulcers, most within the first week, indicating that aphthous ulceration is a common postoperative event. Although this prevalence is comparable to that reported for idiopathic recurrent aphthous stomatitis (RAS) in the general population [3], the temporal clustering after surgery suggests that surgical trauma is associated with ulcer development in susceptible individuals rather than directly causing de novo lesions [31]. While ulcers were generally self-limiting, they negatively affected postoperative comfort, oral function, and quality of life [7] with potential implications for patient satisfaction and adherence to periodontal care.

A key methodological consideration is the distinction between aphthous ulcers and surgically induced traumatic ulcers. The anatomical distribution observed, 88% of lesions on non-keratinized buccal mucosa at sites distant from the surgical field, supports their classification as aphthous ulcers rather than traumatic lesions, which typically occur directly on surgical sites or adjacent to sutures. This distinction, combined with application of validated diagnostic criteria, strengthens the validity of our outcome classification

To clarify who is most at risk, two CHAID decision-tree models were developed. Model 1 (73.6% accuracy) predicted a history of pre- surgical RAS and identified age, marital status, and smoking as key predictors: younger age was associated with higher RAS prevalence [15], marital status likely reflects psychosocial and lifestyle differences [17] and smoking appeared protective, possibly via mucosal keratinization [18] suggesting idiopathic RAS involves age-related physiology, behavior, and psychosocial factors [10] In contrast, Model 2 (79.4% accuracy) identified only age and family history as predictors of post- surgical ulcers, with behavioral factors such as smoking absent. The latter difference implies that intrinsic biological susceptibility, enhanced inflammatory reactions in younger patients, are more likely to be linked to post-surgical ulceration [3], and genetic predispositions revealed by family history [32] rather than by chronic exposures.

The CHAID method has clinical benefits compared with the conventional regression method since it forms explicit decision rule of risk stratification. To illustrate, young patients who are less than 45 years old and have a family history of RAS are a high-risk group that can be pre-empted by counseling and prevention measures. Nonetheless, these models are to be validated in independent samples prior to clinical use because the accuracy estimates of such models could be overly optimistic because of overfitting.

These results correspond to accepted pathophysiological principles. Periodontal surgery destabilizes the mucosal barrier and causes the inflammatory cascade with the activation of immune system, cytokine release, and remodeling of tissues [33] In genetically or immunologically predisposed individuals, these responses may exceed the threshold for ulcer formation [3,32,34]. Postoperative microbiome alterations and perioperative psychological stress may further amplify inflammatory responses and mucosal susceptibility [14,17].

Several unmeasured factors may have influenced our results. First, surgical duration and degree of tissue trauma were not quantified and may confound the relationship between procedure type and ulcer outcomes. Second, perioperative psychological stress, a known trigger for RAS, was not assessed using validated instruments. Third, nutritional status (particularly iron, vitamin B12, and folate deficiencies) was not evaluated, despite established associations with RAS. Fourth, suturing technique and tension could influence mucosal irritation. Fifth, subclinical autoimmune conditions or medication effects (e.g., beta-blockers for hypertension) may have contributed to ulcer susceptibility in some patients [35]. These unmeasured confounders should be addressed in future studies.

Surgical-related factors also influenced outcomes. More complex procedures were significantly associated with broader medication regimens, suture selection, and a higher number of ulcers, whereas ulcer size and anatomical distribution—most commonly minor lesions on the buccal mucosa were not procedure dependent. This suggests that surgical complexity increases ulcer burden but not ulcer severity, which appears to be largely determined by patient-level immune factors [3,27].

The association between procedure type and ulcer occurrence approached but did not reach statistical significance (*p* = 0.065), indicating a trend that requires confirmation in larger samples. The significant association with ulcer number suggests that when ulcers occur, complex procedures are associated with higher lesion counts, even if the overall occurrence rate is not statistically different.

Collectively, these results support a targeted, patient-centered approach to postoperative ulcer prevention and management. Screening for age and personal or family history of aphthous ulcers, appropriate preoperative counseling, and selective use of preventive measures such as topical corticosteroids, chlorhexidine, and atraumatic surgical techniques may reduce postoperative morbidity and improve patient experience [15,36].

### Strengths and Limitations

The study advantages are standardized prospective data collection, a relatively large and varied surgical cohort, assessment by calibrated examiners, and non-linear risk profile identification using CHAID analysis [30].

This paper presents some limitations applicable to the study results. The cross-sectional study and single-center location do not allow for making causal conclusions and generalizations. Incidence of ulcers cannot be compared to baseline because of the absence of a non-surgical control group. Limitations of measurement consist of the possibility of misclassifying between the types of ulcers, the unmeasured confounding variables such as the duration of surgery and psychological pressure, and the failure to measure some of the effects of medications. Analytical drawbacks include unproven CHAID models and underpowered subgroup analysis. There was a two-week follow-up, which posed a risk of missing ulcers, and genetic susceptibility was determined based on family history and not through direct analysis.

Genetic, biomarker, microbiome, nutritional, and patient-reported outcome measures should be introduced into future multicenter and longitudinal studies, and validated tools of stress assessment and comprehensive surgical characterization should be applied to ensure the validation of such predictors and the optimization of preventive measures.

## 5. Conclusions

Postoperative aphthous ulceration occurs in approximately one-fifth of patients undergoing periodontal surgery and represents a clinically relevant postoperative event. Using CHAID decision tree analysis, this study identified distinct risk profiles for preoperative ulcer history and postoperative ulcer development, highlighting the roles of age, personal and family history, and surgical complexity. These findings support the use of structured preoperative risk assessment and targeted preventive strategies to optimize patient-centered periodontal surgical care.

## Figures and Tables

**Table 1:** Demographic and Clinical Characteristics of Study Population (N = 227).

Characteristic	Category	*n* (%)
Age Group	18–25 years	18 (7.9%)
	26–35 years	45 (19.8%)
	36–45 years	52 (22.9%)
	46–55 years	41 (18.1%)
	56–65 years	44 (19.4%)
	≥65 years	22 (9.7%)
Gender	Male	96 (42.3%)
	Female	131 (57.7%)
Marital Status	Married	175 (77.1%)
	Single	52 (22.9%)
Medical Conditions	None reported	182 (80.2%)
	Hypertension	12 (5.3%)
	Type II Diabetes Mellitus	10 (4.4%)
	Hypothyroidism	5 (2.2%)
	Asthma	4 (1.8%)
	Cardiovascular disease	3 (1.3%)
	Other	11 (4.8%)
Current Medications	None	180 (79.3%)
	Beta-blockers	7 (3.1%)
	ACE inhibitors	5 (2.2%)
	Calcium channel blockers	4 (1.8%)
	Levothyroxine	5 (2.2%)
	Oral hypoglycemics	8 (3.5%)
	Other	18 (7.9%)
Family History of Oral Ulcers	Yes	5 (2.2%)
	No	222 (97.8%)
Personal History of Oral Ulcers	Yes	42 (18.5%)
	No	185 (81.5%)
Smoking Status	Current smoker	42 (18.5%)
	Non-smoker	185 (81.5%)

**Table 2:** Summary of CHAID Decision Tree Models for Oral Ulceration Outcomes before and after Procedure.

Characteristic	Model 1: Patient History of Oral Ulcers Before Procedure	Model 2: Patient Experience Ulceration After Procedure
Model Specifications		
Growing method	CHAID	CHAID
Dependent variable	Patient history of oral ulcers	Patient experience ulceration after the procedure
Independent variables tested	Gender, marital status, female patient pregnant, citizenship, education level, occupation, patients condition, allergies, current medications, family history of oral ulcers, patient’s smoke, number of cigarettes patient smoke per day, age	Gender, marital status, female patient pregnant, citizenship, education level, occupation, patients condition, allergies, current medications, family history of oral ulcers, patient’s smoke, number of cigarettes patient smoke per day, age
Validation	None	None
Maximum tree depth	3	3
Minimum cases in parent node	100	100
Minimum cases in child node	50	50
Model Results		
Independent variables included	Age, marital status, patient’s smoke	Age, family history of oral ulcers
Number of nodes	7	5
Number of terminal nodes	4	3
Tree depth	2	2
Risk estimate	0.264	0.206
Risk *SE*	0.029	0.027
Overall classification accuracy	73.6%	79.4%

Note. CHAID = Chi-squared Automatic Interaction Detection. Model 1 examined predictors and risk factors of a patient’s history of oral ulcers before the procedure. Model 2 examined predictors of post-procedure ulceration.

**Table 3:** Chi-Square Analysis of Periodontal Procedure of Patient Undergoing by Clinical Variables.

Clinical Variable and Categories	None (*n* = 9)	GBR (*n* = 30)	Periodontal Flap (*n* = 28)	Soft Tissue Aug (*n* = 50)	Implant Surgery (*n* = 50)	Tooth Extraction (*n* = 13)	Resective Surgery (*n* = 13)	Combined Procedures * (*n* = 34)
Medication After Procedure								
None	6 (66.7%)	9 (30.0%)	13 (46.4%)	21 (42.0%)	8 (16.0%)	5 (38.5%)	6 (46.2%)	5 (14.7%)
Antibiotics	0 (0.0%)	1 (3.3%)	0 (0.0%)	0 (0.0%)	2 (4.0%)	0 (0.0%)	0 (0.0%)	0 (0.0%)
NSAID	1 (11.1%)	0 (0.0%)	9 (32.1%)	12 (24.0%)	12 (24.0%)	4 (30.8%)	0 (0.0%)	0 (0.0%)
Chlorhexidine mouthwash	0 (0.0%)	0 (0.0%)	1 (3.6%)	1 (2.0%)	0 (0.0%)	0 (0.0%)	0 (0.0%)	0 (0.0%)
Antibiotics + NSAID	2 (22.2%)	3 (10.0%)	0 (0.0%)	2 (4.0%)	10 (20.0%)	2 (15.4%)	0 (0.0%)	1 (2.9%)
NSAID + Corticosteroids	0 (0.0%)	0 (0.0%)	0 (0.0%)	1 (2.0%)	0 (0.0%)	0 (0.0%)	0 (0.0%)	0 (0.0%)
NSAID + Chlorhexidine	0 (0.0%)	1 (3.3%)	4 (14.3%)	10 (20.0%)	1 (2.0%)	2 (15.4%)	7 (53.8%)	2 (5.9%)
Antibiotics + NSAID + Corticosteroids	0 (0.0%)	10 (33.3%)	0 (0.0%)	0 (0.0%)	2 (4.0%)	0 (0.0%)	0 (0.0%)	2 (5.9%)
Antibiotics + NSAID + Chlorhexidine	0 (0.0%)	2 (6.7%)	0 (0.0%)	3 (6.0%)	12 (24.0%)	0 (0.0%)	0 (0.0%)	18 (52.9%)
Antibiotics + Corticosteroids + Chlorhexidine	0 (0.0%)	1 (3.3%)	0 (0.0%)	0 (0.0%)	2 (4.0%)	0 (0.0%)	0 (0.0%)	4 (11.8%)
NSAID + Corticosteroids + Chlorhexidine	0 (0.0%)	0 (0.0%)	1 (3.6%)	0 (0.0%)	1 (2.0%)	0 (0.0%)	0 (0.0%)	0 (0.0%)
All Above	0 (0.0%)	3 (10.0%)	0 (0.0%)	0 (0.0%)	0 (0.0%)	0 (0.0%)	0 (0.0%)	2 (5.9%)
Suture Type								
Vicryl	6 (66.7%)	12 (40.0%)	23 (82.1%)	27 (54.0%)	39 (78.0%)	10 (76.9%)	4 (30.8%)	17 (50.0%)
PTFE	0 (0.0%)	6 (20.0%)	1 (3.6%)	2 (4.0%)	2 (4.0%)	0 (0.0%)	2 (15.4%)	6 (17.6%)
Prolene	3 (33.3%)	12 (40.0%)	4 (14.3%)	17 (34.0%)	9 (18.0%)	3 (23.1%)	7 (53.8%)	11 (32.4%)
Monocryl	0 (0.0%)	0 (0.0%)	0 (0.0%)	4 (8.0%)	0 (0.0%)	0 (0.0%)	0 (0.0%)	0 (0.0%)
Patient Experience Ulceration								
Yes	0 (0.0%)	6 (20.0%)	7 (25.0%)	8 (16.0%)	8 (16.0%)	2 (15.4%)	1 (7.7%)	15 (44.1%)
No	9 (100%)	24 (80.0%)	21 (75.0%)	42 (84.0%)	42 (84.0%)	11 (84.6%)	12 (92.3%)	19 (55.9%)
Number of Ulcers								
None	9 (100%)	24 (80.0%)	21 (75.0%)	42 (84.0%)	42 (84.0%)	11 (84.6%)	12 (92.3%)	19 (55.9%)
1–5 ulcers	0 (0.0%)	6 (20.0%)	7 (25.0%)	8 (16.0%)	8 (16.0%)	1 (7.7%)	1 (7.7%)	14 (41.2%)
6–10 ulcers	0 (0.0%)	0 (0.0%)	0 (0.0%)	0 (0.0%)	0 (0.0%)	1 (7.7%)	0 (0.0%)	1 (2.9%)
Ulcer/Ulcers Size								
None	9 (100%)	24 (80.0%)	21 (75.0%)	42 (84.0%)	42 (84.0%)	11 (84.6%)	12 (92.3%)	21 (61.8%)
3–10 mm (Minor)	0 (0.0%)	5 (16.7%)	6 (21.4%)	8 (16.0%)	6 (12.0%)	2 (15.4%)	1 (7.7%)	12 (35.3%)
1–3 cm (Major)	0 (0.0%)	1 (3.3%)	1 (3.6%)	0 (0.0%)	2 (4.0%)	0 (0.0%)	0 (0.0%)	1 (2.9%)
None	9 (100%)	24 (80.0%)	21 (75.0%)	44 (88.0%)	45 (90.0%)	11 (84.6%)	13 (100%)	25 (73.5%)
Right Ventral tongue	0 (0.0%)	0 (0.0%)	1 (3.6%)	0 (0.0%)	0 (0.0%)	0 (0.0%)	0 (0.0%)	0 (0.0%)
Right Lateral tongue	0 (0.0%)	0 (0.0%)	0 (0.0%)	0 (0.0%)	1 (2.0%)	0 (0.0%)	0 (0.0%)	1 (2.9%)
Left Lateral tongue	0 (0.0%)	0 (0.0%)	0 (0.0%)	0 (0.0%)	0 (0.0%)	0 (0.0%)	0 (0.0%)	1 (2.9%)
Right Buccal mucosa	0 (0.0%)	4 (13.3%)	6 (21.4%)	1 (2.0%)	2 (4.0%)	2 (15.4%)	0 (0.0%)	5 (14.7%)
Left Buccal mucosa	0 (0.0%)	0 (0.0%)	0 (0.0%)	0 (0.0%)	1 (2.0%)	0 (0.0%)	0 (0.0%)	0 (0.0%)
Right Labial mucosa	0 (0.0%)	0 (0.0%)	0 (0.0%)	3 (6.0%)	0 (0.0%)	0 (0.0%)	0 (0.0%)	1 (2.9%)
Left Ventral & Lateral tongue	0 (0.0%)	1 (3.3%)	0 (0.0%)	1 (2.0%)	0 (0.0%)	0 (0.0%)	0 (0.0%)	0 (0.0%)
Right & Left Labial mucosa	0 (0.0%)	0 (0.0%)	0 (0.0%)	1 (2.0%)	0 (0.0%)	0 (0.0%)	0 (0.0%)	0 (0.0%)
Left Floor & Buccal mucosa	0 (0.0%)	0 (0.0%)	0 (0.0%)	0 (0.0%)	1 (2.0%)	0 (0.0%)	0 (0.0%)	0 (0.0%)
Right Floor & Buccal Mucosa	0 (0.0%)	1 (3.3%)	0 (0.0%)	0 (0.0%)	0 (0.0%)	0 (0.0%)	0 (0.0%)	0 (0.0%)
Right & Left Buccal mucosa	0 (0.0%)	0 (0.0%)	0 (0.0%)	0 (0.0%)	0 (0.0%)	0 (0.0%)	0 (0.0%)	1 (2.9%)

Note. * Combined procedures include: GBR and Periodontal flap surgery (n = 4), GBR and Soft tissue augmentation (n = 2), GBR and Implant surgery (n = 7), GBR and Tooth extraction (n = 6), Soft tissue augmentation and Implant surgery (n = 2), Soft tissue augmentation and Tooth extraction (n = 1), Immediate implant surgery and Tooth extraction (n = 6), GBR + Soft tissue augmentation + Implant surgery (n = 1), GBR + Implant surgery + Tooth extraction (n = 5).

**Table 4:** Chi-Square Analysis of Periodontal Procedure Type by Clinical Variables (Abbreviated).

Variable	χ^2^ Value	df	*p*-Value
Medication After Procedure	300.45	77	<0.001 **
Suture Type	69.19	21	0.024 *
Patient Experience Ulceration	25.27	7	0.065
Number of Ulcers	48.43	14	0.031 *
Ulcer Size	33.26	14	0.406
Ulcer Location	168.61	77	0.642

Note. Medication After Procedure (χ^2^ = 300.45, *p* ≤ 0.001 **), Suture Type (χ^2^ = 69.19, *p* = 0.024 *), Patient Experience Ulceration (χ^2^ = 25.27, *p* = 0.065), Number of Ulcers (χ^2^ = 48.43, *p* = 0.031 *), Ulcer/Ulcers Size (χ^2^ = 33.26, *p* = 0.406), Ulcer Location by Anatomical Site (χ^2^ = 168.61, *p* = 0.642). * *p* < 0.05. ** *p* < 0.001.

## Data Availability

Data are available and can be obtained from the corresponding author upon reasonable request.

## Abbreviations

The following abbreviations are used in this manuscript:

RAS	recurrent aphthous stomatitis
